# Influence of fluid balance on postoperative outcomes after hepatic resection in patients with left ventricular diastolic dysfunction

**DOI:** 10.3389/fsurg.2022.1036850

**Published:** 2022-11-16

**Authors:** Jungho Shin, Suk-Won Suh

**Affiliations:** ^1^ Department of Internal Medicine, College of Medicine, Chung-Ang University, Seoul, Korea; ^2^ Department of Surgery, College of Medicine, Chung-Ang University, Seoul, Korea

**Keywords:** left ventricular diastolic dysfunction, fluid balance, acute kidney injury, pulmonary edema or effusion, hepatic resection

## Abstract

**Objective:**

The maintenance of low central venous pressure (CVP) during hepatic resection is associated with a reduction in estimated blood loss. After completion of the hepatic parenchymal transection, fluid is rapidly administered to replace the surgical blood loss and fluid deficit to prevent subsequent organ injury risk. However, this perioperative fluid strategy may influence on the postoperative outcomes of patients with left ventricular diastolic dysfunction (LVDD) who cannot tolerate volume adjustment.

**Method:**

A total of 206 patients with who underwent hepatic resection between March 2015 and February 2021 were evaluated. LVDD was defined according to the American Society of Echocardiography and the European Association of Cardiovascular Imaging 2016 recommendations as LVDD (group A, *n* = 39), or normal LV diastolic function and indeterminate decision (group B, *n* = 153). We compared the clinical outcomes of patients between two groups, and then analyzed the risk factors for postoperative complications.

**Result:**

Postoperative acute kidney injury (AKI, 10.3% vs. 1.3%, *P* = 0.004) and pleural effusion or edema (51.3% vs. 30.1%, *P* = 0.013) were more common in group A than in group B. Further, creatinine levels from postoperative day 1 to day 7 were significantly higher and daily urine outputs at postoperative day 1 (*P* = 0.038) and day 2 (*P* = 0.025) were significantly lower in group A than in group B. LVDD was the only significant risk factor for postoperative AKI after hepatic resection (odds ratio, 10.181; 95% confidence interval, 1.570–66.011, *P* = 0.015).

**Conclusions:**

The rates of renal dysfunction and pulmonary complications after hepatic resection are higher in patients with LVDD than in those with normal LV diastolic function. Thus, these patients require individualized fluid management.

## Introduction

Despite recent advances in surgical techniques, blood loss during hepatic resection remains a major concern that significantly influences postoperative morbidity and mortality (1). Given the different inflow and outflow systems of the hepatic vasculature, bleeding from the hepatic vein, which directly drains into the inferior vena cava, can be massive and difficult to control (2). Maintaining a low central venous pressure (CVP) during hepatic resection has emerged as an effective strategy to minimize intraoperative blood loss (3, 4). Hepatic blood congestion, induced by elevated CVP, leads to an incremental increase in transmural pressure and distension of hepatic veins. These veins are consequently torn easily, promoting blood loss at the time of parenchymal transection. Preoperative fluid restriction is one of the most effective and commonly used methods for lowering CVP (5). After completion of the hepatic parenchymal transection, fluid is rapidly administrated to replace the surgical blood loss and fluid deficit to prevent the relative hypotension and potential hypoperfusion of abdominal organs during hepatic resection, which may lead to dysfunction in the postoperative period (6).

Left ventricular diastolic dysfunction (LVDD) is characterized by abnormal myocardial relaxation and filling during diastole and subsequently increased left ventricular (LV) filling pressure (7). Patients with LVDD present with diminished LV compliance that can be intolerable to volume adjustments. Intravenous fluid administration in these patients is associated with an increased risk of fluid overload that is in turn associated with postoperative morbidity and mortality (8). Previous studies reported that LVDD may have adverse impacts on renal function and mortality in sepsis patients who require intravenous fluid administration to maintain organ perfusion (9, 10). The kidneys are encapsulated organs and are thus sensitive to the effects of tissue edema. Accordingly, renal perfusion would be decreased in patients with fluid overload (11). The incidence of major cardiovascular events is also significantly higher after noncardiac surgery in patients with LVDD (12).

We hypothesized that the perioperative fluid management related to maintaining a low CVP during hepatic resection for minimizing intraoperative blood loss might have a negative impact on postoperative outcomes of patients with LVDD who cannot tolerate volume adjustment.

This study aimed to compare the influence of perioperative fluid strategy during hepatic resection on postoperative outcomes in patients with and without LVDD.

## Materials and methods

### Patients

**Figure 1:**
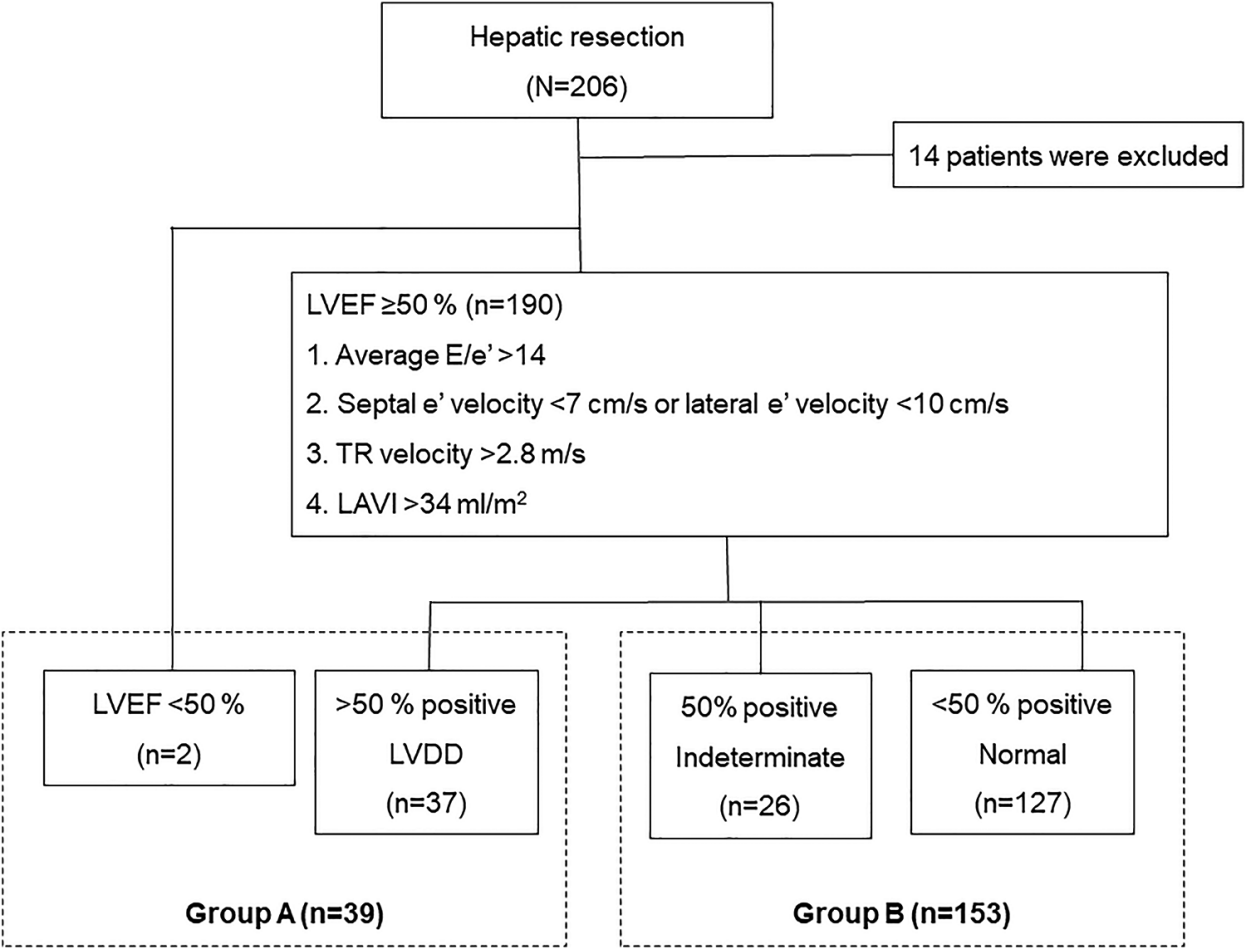
Patient inclusion flowchart. The patients are classified according to their LV diastolic function assessed based on echocardiographic assessment using the ASE and the EACVI 2016 recommendations. LV, left ventricular; ASE, American Society of Echocardiography; EACVI, European Association of Cardiovascular Imaging.

This retrospective study evaluated 206 patients who underwent open liver resection between March 2015 and February 2021 at our hospital. The exclusion criteria were as follows: upper abdominal surgical history (*n* = 2); combined operation (*n* = 4); treatment history of hepatic lesion (*n* = 1); borderline liver function (*n* = 2); preoperative renal dysfunction, defined as a glomerular filtration rate (eGFR) < 90 ml/min (*n* = 4); and insufficient clinical data (*n* = 1). Finally, 192 patients were enrolled in the study and stratified into two groups according to LV diastolic function based on echocardiographic assessment using the American Society of Echocardiography (ASE) and the European Association of Cardiovascular Imaging (EACVI) 2016 recommendations (7): LVDD (group A, *n* = 39), or others including normal LV diastolic function and indeterminate decision (group B, *n* = 153; Figure 1). We compared the clinical outcomes in patients with and without LVDD, and then analyzed the risk factors for postoperative complications.

We reported our findings according to the Strengthening the Reporting of Observational studies in Epidemiology (STROBE) guidelines (13). This study was approved by our Institutional Review Board (IRB No. 2108-009-19379) and was performed in accordance with the Declaration of Helsinki (as revised in 2013). The need for informed consent was waived because accrual patient records were analyzed, and no patient identification data were used.

### Echocardiography

Preoperative transthoracic echocardiography was performed by sonographers or cardiologists using Philips CX50 (Philips Medical Systems, Bothell, WA, United States), and the findings were interpreted by board-certified cardiologists. Parameters were obtained using comprehensive M-mode two-dimensional Doppler echocardiography from the long- and short-axis parasternal views; apical four-chamber, two-chamber, and long-axis views; and subcostal views. LV ejection fraction (LVEF) was calculated using the biplane approach and modified using Simpson's method. The peak velocity of the transmitral inflow waveform during early (E) and late (A) diastole was recorded at the tip of the valvular leaflets. The peak velocities were measured at early diastole (septal e' and lateral e’), and the values were averaged (averaged e') at the septal and lateral mitral annulus. The peak velocity of tricuspid regurgitation flow (TR velocity) was determined using the continuous Doppler method. Left atrial maximum volume at left ventricular end-systole (LAV) was computed using Simpson's method and was indexed by body surface area (LAVI).

The patients with preserved LVEF were classified according to their LV diastolic function based on echocardiographic assessment using the ASE and the EACVI 2016 recommendations (7): average E/e' > 14; septal e' velocity <7 cm/s or lateral e' velocity <10 cm/s, TR velocity >2.8 m/s, and LAVI >34 ml/m^2^. Patients with >50% of these findings were diagnosed with LVDD, while patients with <50% of these findings were considered to have normal left ventricular diastolic function. The presence of 50% positive findings resulted in an intermediate decision.

### Data collection

Clinico-demographic data, including age, sex, body mass index, presence of diabetes mellitus, hypertension, hepatitis B virus (HBV) or hepatitis C virus (HCV), American Society of Anesthesiology (ASA) score, and diagnosis, were collected. Type of hepatic resection was classified into major resection, defined as resection of three or more segments, or minor resection. Operative data including operative duration, amount of fluids administered, vasopressor (ephedrine or phenylephrine) usage and their total amount, requirement of blood transfusion, urine output, and estimated blood loss were investigated. Perioperative laboratory results of liver function (e.g., total bilirubin [TB], international normalized ratio [INR], and albumin) and renal function (e.g., creatinine at admission; operative day; and postoperative days 1, 3, 5, and 7) were analyzed. In addition, all available intake records, composed of oral and parenteral fluids and output data including urine, gastrointestinal losses, and drains, from operative day to postoperative day 7 were collected. Positive fluid balance was defined as the amount of positive input more than 12 ml/kg/h during the operation and 1.5 ml/kg/h in the 24 h postoperative period (14). Data regarding the nature and incidence of postoperative complications, intensive care unit (ICU) admission, and length of postoperative hospitalization were also collected. Acute kidney injury (AKI) was defined in accordance with the 2012 Kidney Disease Improving Global Outcomes guidelines (15) which had higher predictability than other criteria for assessing prognosis (16): increase in serum creatinine by ≥0.3 mg/dl within 48 h; increase in serum creatinine to ≥1.5 times baseline within 7 days before surgery; or urine volume <0.5 ml/kg/h for 6 h. Pulmonary effusion or edema was diagnosed by radiologist that showed a costophrenic angle blunting with abnormal accumulation of fluid in the extravascular compartments of the lung on x-ray findings in postoperative period. We used a term pulmonary effusion or edema because it cannot be distinguished without clinical evaluation. Myocardial infarction was determined by chest symptoms and ST changes on electrocardiogram and/or elevated cardiac troponin I. Postoperative liver insufficiency was defined as a peak postoperative TB level of >7 mg/dl and/or the presence of ascites >500 ml/day based on a previous study (17).

### Anesthetic and surgical technique

Anesthetic management was performed using a standard protocol in our hospital. General anesthesia was induced with intravenous 100 μg of fentanyl and 1.2 mg/kg of propofol, followed by intravenous 1 mg/kg of rocuronium to facilitate endotracheal tube placement. General anesthesia was maintained with sevoflurane (2 to 3 volume%), nitrous oxide (1.8 L/min), and O2 (1.2 L/min). Intravenous 1 mg/kg Rocuronium was administered, as required, to maintain adequate surgical relaxation. All patients underwent ultrasonography-guided right internal jugular vein catheterization after tracheal intubation in the operating room, and the position of the catheter was determined using a chest radiograph. Electrocardiogram, pulse oximetry, end-tidal carbon dioxide, invasive radial arterial pressure, CVP, and urine output were monitored. Fluid was not administered preoperatively and was restrictively infused after the start of anesthesia, maintaining CVP less than 5 mmHg until the hepatic parenchymal transection was complete. Thereafter, the crystalloid fluid was rapidly infused at 10 to 12 ml/kg/h to replace the surgical blood loss or fluid deficit, including insensible loss during the operation, and a colloid solution, hydroxyethyl-starch (HES), was used considering volume status in the operating room. We used vasopressor drug when the MAP decreased below 60 mmHg. Mostly 5 mg bolus of ephedrine was administered, but if an elevated heart rate was present, 50 mcg bolus of phenylephrine were injected. Red blood cells were transfused considering estimated blood loss and level of hemoglobin concentration (maintain ≥8 g/dl) in the operative room and if the hemoglobin concentration decreased to <8 g/dl in the postoperative period. ICU admission was decided if the patients required inotropic agents or had cardiac arrhythmia during the operation.

All hepatic resections were performed by one surgeon using the same hepatic parenchymal transection technique. The extent of hepatic resection was determined based on tumor size and location. Parenchymal transection was performed using an ultrasonic aspirator, metal clips, and electrocautery device, and the cutting surface of the liver was sprayed with biological glue.

### Statistical analysis

For intergroup comparisons, the data distribution was initially evaluated for normality using the Shapiro–Wilk test. Normally distributed data were presented as means±standard deviations, and between-group comparisons were conducted using Student's *t*-test or Kruskal–Wallis test. Meanwhile, between-group comparisons of descriptive data were conducted using the *χ*2 test and Fisher's exact test. Multivariate analysis using an ordinary logistic regression model was performed to investigate the risk factors for specific postoperative morbidities. All statistical analyses were conducted using SPSS Statistics for Windows, version 19.0 (IBM Corp., Armonk, NY, United States).

## Results

### Patient characteristics

The median age (67 [50–87] years vs. 54 [22–82] years, *P* < 0.001) and age ≥ 60 (74.4% vs. 38.6%, *P* < 0.001) were significantly increased in group A than in group B. Hypertension was also significantly more prevalent in group A than in group B (69.2% vs. 37.9%, *P* < 0.001). There were no significant differences in sex distribution, body mass index, prevalence of diabetes mellitus, presence of HBV or HCV, ASA score, diagnosis, and baseline liver function (TB, albumin, and INR) and renal function (creatinine level) between the two groups. Major resections, such as right hemi-hepatectomy, extended right hemi-hepatectomy, left hemi-hepatectomy, extended left hemi-hepatectomy, and central hepatectomy, were more significantly more frequent in group B than in group A (38.5% vs. 63.4%, *P* = 0.005; Table 1).

**Table 1:** Clinico-demographic patient characteristics.

	Group A (*n* = 39)	Group B (*n* = 153)	*P*
Age (years), median (range)	67 (50–87)	54 (22–82)	<0.001
Age ≥60	29 (74.4%)	59 (38.6%)	<0.001
Sex (male)	27 (69.2%)	98 (64.1%)	0.545
Body mass index, kg/m^2^	25.2 ± 3.2	25.1 ± 5.7	0.957
Diabetes mellitus	11 (28.2%)	27 (17.6%)	0.145
Hypertension	27 (69.2%)	57 (37.9%)	<0.001
Presence of HBV	13 (33.3%)	39 (25.5%)	0.337
Presence of HCV	0 (0%)	3 (2.0%)	0.377
ASA score			
I	32 (86.5%)	134 (86.5%)	
II	4 (10.8%)	19 (12.3%)	
III	1 (2.7%)	2 (1.3%)	0.805
Diagnosis			
Hepatocellular carcinoma	24 (61.5%)	102 (66.7%)	
Cholangiocarcinoma	4 (10.3%)	7 (4.6%)	
Colorectal liver metastasis	9 (23.1%)	28 (18.3%)	
Other benign liver disease	2 (5.1%)	16 (10.5%)	0.364
Echocardiographic findings			
TR degree (moderate to severe)	6 (15.4%)	22 (15.7%)	0.687
TRPG ≥30 mmHg	12	40	0.565
Baseline liver function			
Total bilirubin	0.7 ± 0.3	0.6 ± 0.2	0.215
Albumin	4.2 ± 0.4	4.2 ± 0.5	0.656
INR	1.07 ± 0.08	1.08 ± 0.09	0.363
Baseline renal function			
Creatinine	0.81 ± 0.27	0.74 ± 0.20	0.079
Procedure, *n* (%)			
Major resection (≥3 segments)	15 (38.5%)	97 (63.4%)	
Minor resection (<3 segments)	24 (61.5%)	56 (36.6%)	0.005

Data are presented as the mean±standard deviations or *n* (%) unless otherwise indicated.

HBV, hepatitis B virus; HCV, hepatitis C virus; ASA score, American Society of Anesthesiology score; TR, tricuspid valve regurgitation; TRPG, tricuspid valve pressure gradient; INR, International normalized ratio.

### Operative outcomes

The mean operative duration was significantly longer in group B than in group A (187.4 ± 79.7 min vs. 238.3 ± 91.3 min, *P* = 0.002). There were no significant between-group differences in the intraoperative fluid administration of crystalloid and colloid, amount of urine output, estimated blood loss, fluid balance, and proportion of positive fluid balance. Requirements of vasopressor and its dosage or blood transfusion and transfused red blood cell units showed no statistical differences. There was no significant difference in the ICU admission rate. No patient died. Postoperative hospital stay was longer in group A than in group B (13.6 ± 6.4 days vs. 12.4 ± 7.0 days, *P* = 0.307), but the difference was not significant (Table 2).

**Table 2:** Intraoperative and postoperative patient outcomes.

	Group A (*n* = 39)	Group B (*n* = 153)	*P*
Operative duration, min	187.4 (±79.7)	238.3 (±91.3)	0.002
Intraoperative fluids, ml/kg/hr	12.5 (±6.8)	13.1 (±10.2)	0.127
Crystalloid, ml/kg/hr	10.7 (±6.6)	11.6 (±9.8)	0.593
Colloid, ml/kg/hr	1.7 (±1.2)	1.8 (±1.5)	0.438
Urine output, ml/kg/hr	2.0 (±2.9)	2.1 (±1.9)	0.593
Estimated blood loss, ml	503 (±343)	641 (±796)	0.297
Fluid balance, ml/kg/hr	8.8 (±4.7)	9.8 (±5.6)	0.217
Positive fluid balance (%)	5 (13.5%)	22 (14.2%)	0.915
*Vasopressor usage	10 (25.6%)	35 (22.9%)	0.748
*Vasopressor total dosage, ml, median (IQR)	5 (5–6.25)	5 (5–10)	0.230
Blood transfusion	6 (15.4%)	24 (15.7%)	0.936
Red blood cell units, pint, median (IQR)	1 (1–1.5)	1 (1–2)	0.326
Intensive care unit admission	7 (17.9%)	26 (17.0%)	0.916
Mortality	0	0	–
Postoperative hospital stay, days	13.6 (±6.4)	12.4 (±7.0)	0.307

Data are presented as the mean±standard deviations or *n* (%) unless otherwise indicated, IQR, interquartile range.

* Vasopressor, ephedrine or phenylephrine.

**Figure 2:**
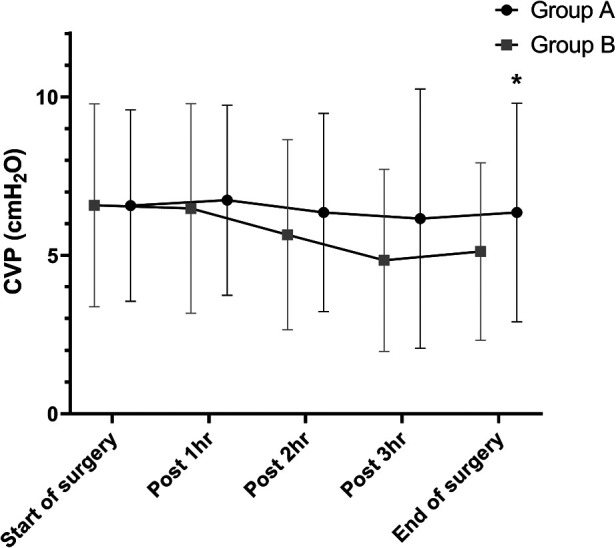
Differences in CVP during hepatic resection. CVPs showed a decreasing trend after the start of surgery until completion of hepatic parenchymal transection and then re-increased after fluid challenge in both groups. There is a significant difference in CVP at the end of surgery between the two groups (6.35 ± 3.45 vs. 5.12 ± 2.80, *P* = 0.025). *P < 0.05 CVP, central venous pressure.

In both groups, CVPs showed a decreasing trend after the start of surgery until completion of hepatic parenchymal transection and then re-increased after fluid challenge. CVP at the end of surgery was significantly between the two groups (6.35 ± 3.45 vs. 5.12 ± 2.80, *P* = 0.025; Figure 2).

### Postoperative outcomes

**Figure 3:**
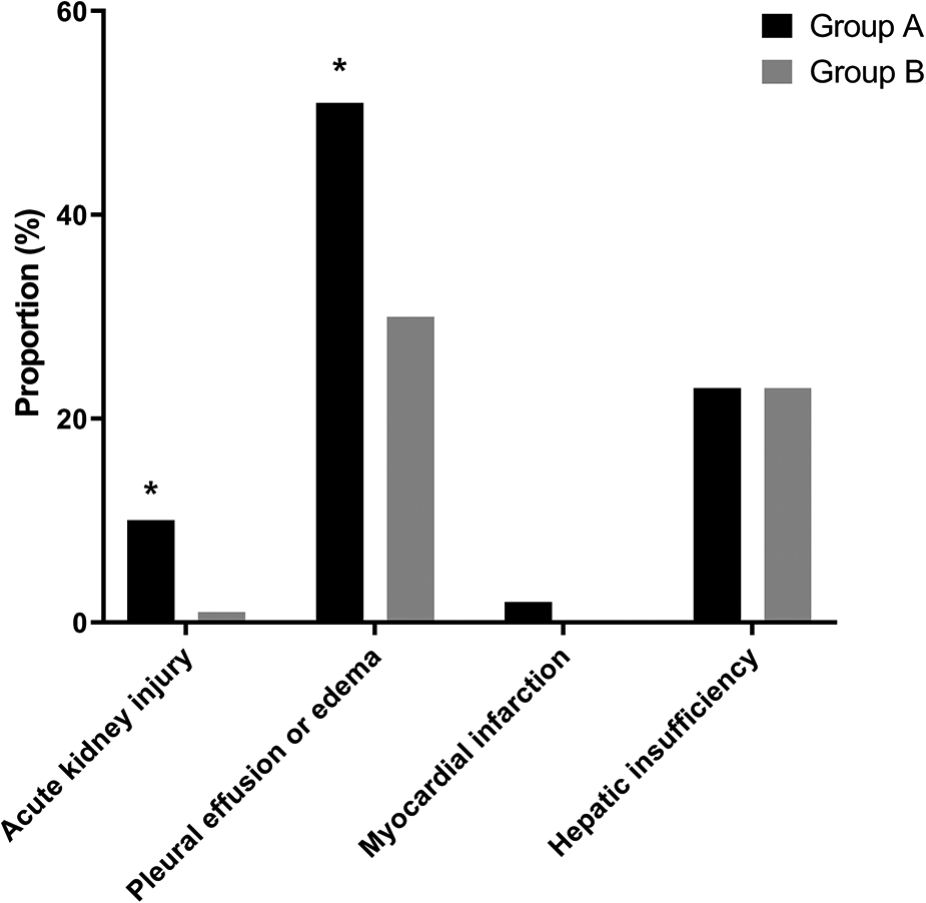
Incidence rates of postoperative complications including acute kidney injury, pulmonary edema or effusion, myocardial infarction, and hepatic insufficiency. Among postoperative complications, the incidence rates of AKI (10.3% vs. 1.3%, *P* = 0.004) and pleural effusion or edema edema (51.3% vs. 30.1%, *P* = 0.013) are significantly higher in the patients with LVDD than in those with Normal LV diastolic function. *P < 0.05, LVDD, left ventricular diastolic dysfunction; LV, left ventricular.

With respect to postoperative complications, the incidence rates of AKI (10.3% vs. 1.3%, *P* = 0.004) and pleural effusion or edema (51.3% vs. 30.1%, *P* = 0.013) were significantly higher in group A than in group B. All postoperative AKIs were classified as stage 1 on the KDIGO classification. Only one patient had myocardial infarction, and the patient was from group A (2.6% vs. 0%, *P* = 0.047). Postoperative hepatic insufficiency was more common in group A than in group B (23.1% vs. 23.5%), but the difference was not significant (*P* = 0.953; Figure 3).

**Figure 4:**
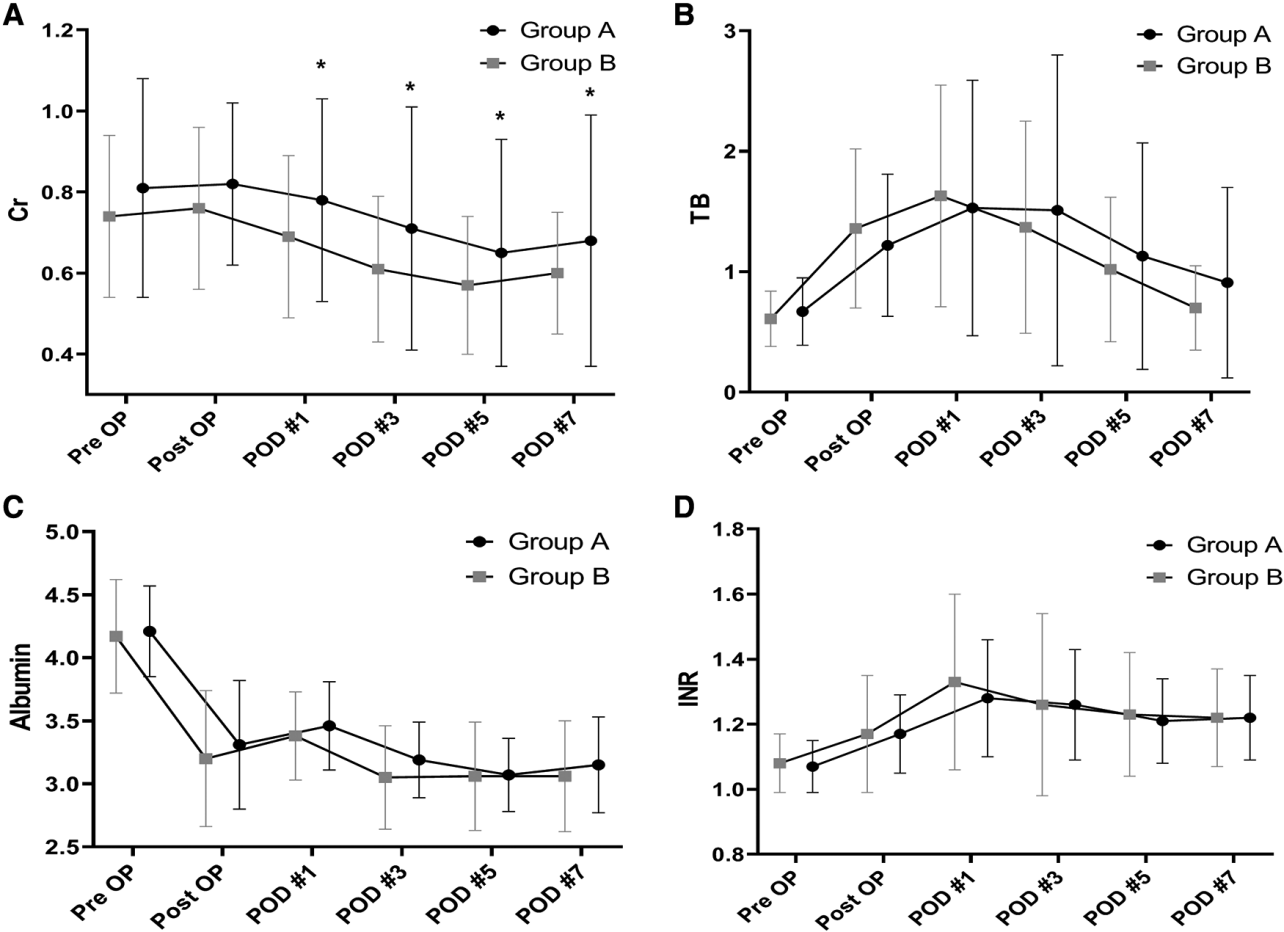
Between-group differences in laboratory results in the perioperative period. Creatinine (**A**). TB (**B**). INR (**C**). Albumin (**D**). Creatinine is significantly higher after postoperative day 1 to day 7in the patients with LVDD than in those with Normal LV diastolic function. *P < 0.05 TB, total bilirubin; INR, international normalized ratio; LVDD, left ventricular diastolic dysfunction; LV, left ventricular.

**Figure 5:**
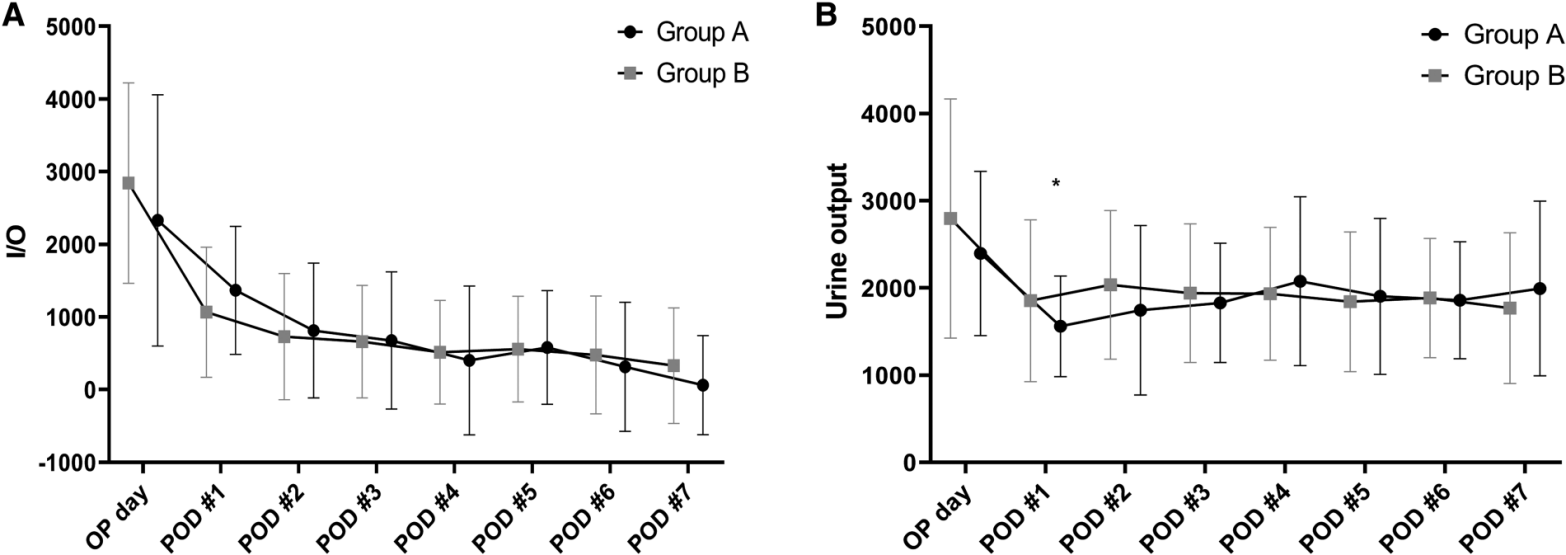
Between-group comparisons in daily fluid balances (**A**) and urine outputs (**B**) in the perioperative period. There are no significant differences in the daily fluid balances between the two groups. However, daily urine outputs are significantly lower in the patients with LVDD than in those with Normal LV diastolic function at postoperative day 1 (1,560 ± 577 vs. 1,853 ± 928, *P* = 0.038) and day 2 (1,744 ± 971 vs. 2,036 ± 853, *P* = 0.024). *P < 0.05, LVDD, left ventricular diastolic dysfunction; LV, left ventricular.

There were no significant between-group differences in TB, INR, and albumin levels as measures of liver function. Creatinine levels were significantly higher in group A than group B from postoperative day 1 to day 7 (Figure 4). Comparison of the daily fluid balances from operative day to postoperative day 7 to identify the differences in fluid management showed no significant differences between the two groups. However, daily urine outputs were significantly lower in group A than in group B at postoperative day 1 (1,560 ± 577 ml/kg/hr vs. 1,853 ± 928 ml/kg/hr, *P* = 0.038) and day 2 (1,744 ± 971 ml/kg/hr vs. 2,036 ± 853 ml/kg/hr, *P* = 0.024; Figure 5).

### Risk factor analysis for postoperative AKI

LVDD was the only significant predictor of AKI in univariate analysis (odds ratio [OR], 8.629; 95% confidence interval [CI], 1.519–49.000, *P* = 0.015) and multivariate analysis (OR, 10.181; 95% CI, 1.570–66.011, *P* = 0.015; Table 3).

**Table 3:** Analysis of risk factors for postoperative acute kidney injury.

Variable	Univariate analysis			Multivariate analysis		
	OR	95% CI	*P*	OR	95% CI	*P*
Age	1.020	0.966–1.076	0.485			
Age ≥ 60	1.800	0.294–11.023	0.525			
Sex (male)	0.360	0.315–24.036	0.360			
BMI	1.067	0.986–1.154	0.108			
Diabetes mellitus	4.314	0.835–22.285	0.081			
Hypertension	2.593	0.463–14.507	0.278			
Presence of HBV	1.360	0.242–7.656	0.727			
Presence of HCV	NS					
ASA score ≥ 2	0.617	0.066–5.749	0.672			
Moderate to severe TR						
TRPG ≥30 mmHg						
Total bilirubin	2.653	0.121–58.212	0.536			
INR	2.029	0.000–8457.360	0.868			
Albumin	0.666	0.112–3.948	0.654			
Creatinine	0.239	0.003–19.011	0.522			
Operation time (min)	1.002	0.994–1.011	0.596			
Major resection	0.706	0.139–3.594	0.675			
Positive fluid balance (intraoperative)	2.500	0.264–23.626	0.424			
Positive fluid balance (postoperative)	4.320	0.687–27.149	0.119			
LVDD	8.629	1.519–49.000	0.015	10.181	1.570–66.011	0.015
Vasopressor usage	0.618	0.070–5.435	0.665			
Transfusion	1.041	0.117–9.245	0.971			
Estimated blood loss	1.000	0.999–1.001	0.807			

HBV, hepatitis B virus; HCV, hepatitis C virus; ASA score, american society of anesthesiology score; TR, tricuspid valve regurgitation; TRPG, tricuspid valve pressure gradient; INR, International normalized ratio; LVDD, left ventricular diastolic dysfunction; OR, odds ratio; CI, confidence interval.

## Discussion

This retrospective study showed that postoperative AKI and pulmonary edema or effusion were more common in patients with LVDD than in those with normal LV diastolic function. Also, this study showed that LVDD was the only significant predictor of postoperative AKI in a multivariate analysis.

Patients with LVDD are vulnerable to changes in fluid status, and lowering preload during an operation could decrease cardiac output, possibly leading to hypoperfusion of abdominal organs, making it a risk factor for postoperative renal dysfunction. An analysis on the intraoperative changes of cardiac stroke volume would reveal any influence on clinical outcomes. However, since this study was retrospective in nature, no such analysis was performed. Patients with LVDD are also at risk of fluid overload, where fluid challenge after completion of hepatic resection can conversely cause postoperative renal dysfunction. Fluid overload is associated with a high risk of AKI and delayed recovery because tissue edema of the kidneys leads to hypoperfusion-induced organ injury (18). Previous studies have shown that patients with LVDD often have elevated CVP; this, in turn, is negatively correlated with eGFR and increases the risk of postoperative AKI (19, 20). In the current study, CVP at the end of surgery with fluid challenge after hepatic parenchymal transection was higher in patients with LVDD. The incidence of postoperative AKI was also significantly higher in these patients than in those with normal LV diastolic function despite a similar fluid balance. In addition, we used CVP to assess the volume status, although it has been shown to have limited accuracy (21). Further studies to confirm these results are required using more reliable indicators reflecting fluid status and responsiveness, such as inferior vena cava size.

The current study showed that patients with LVDD had significantly lower daily urine outputs in the early postoperative period than those with normal LV diastolic function. Fluid therapy in this period is important to avoid fluid overload, which is associated with the development of AKI. We adjusted the amounts of postoperative fluid administration according to urine outputs so that there were no significant differences in daily fluid balances between the two groups. This adjustment might have influenced the lack of significant difference in postoperative hospital stay between patients with and without LVDD. All cases of postoperative AKIs were classified as stage 1 on the KDIGO classification, where they were resolved at discharge without requiring renal replacement therapy. However, AKI was associated with prolonged postoperative hospital stay (22.0 ± 14.3 days vs. 12.3 ± 6.3 days, *P* = 0.001). Thus, the use of nephrotoxic medications, particularly those that cause either glomerular or interstitial damage, should be avoided in patients with LVDD, especially in the early postoperative period.

LVDD is associated with adverse postoperative cardiovascular events (22). Previous studies showed that LVDD is an independent risk factor for postoperative pulmonary edema in patients undergoing non-cardiac surgery due to the increase in LV filling pressure concomitant with pulmonary venous pressure (23). The current study also found that pleural effusion or pulmonary edema was significantly higher in patients with LVDD than those with normal diastolic function. Although most cases of pleural effusion or pulmonary edema were spontaneously improved with or without diuretics, two patients with LVDD (5.1%) required percutaneous drainage because of the increasing oxygen requirement. LVDD can also impair coronary flow reserve, increasing LV wall stress in patients with normal diastolic function and leading to increased myocardial blood flow (24). Patients with LVDD are at risk of coronary flow reduction after volume adjustment, resulting in myocardial ischemia and adverse cardiovascular events. In the current study, one patient with LVDD had a myocardial infarction requiring a stent placement on postoperative day 1.

Various methods for monitoring cardiac preload and fluid volume were reported in previous studies. Intraoperative monitoring of stroke volume variation (SVV) can be measured non-invasively using the FloTrac/Vigileo system (Edwards Lifesciences, Irvine, Calif, United States), which has been shown to have high sensitivity and specificity for predicting a patient's volume status and is not influenced by patient positioning or mechanical ventilation (25). One study suggested an SVV-based goal-directed therapy (GDT) protocol for reducing blood loss during hepatectomy (26). The GDT protocol incorporates three hemodynamic components, SVV, cardiac index, and MAP, and these parameters are used according to the hepatectomy phase. Bioelectrical impedance analysis (BIA) is another practical method that has been used in several studies assessing volume status in critically ill patients and has the advantages of noninvasiveness, rapid processing, and easy handling (27). BIA quantifies the human body composition, and the ratio of extracellular water to total body water (ECW/TBW) can be calculated as an index of volume status. This index is elevated in patients with volume overload because excess volume primarily accumulates in the ECW. We previously suggested that BIA for preoperative volume assessment during hepatic resection can be utilized to maintain a low CVP and guide fluid management intraoperatively and postoperatively (28).

There are several limitations to this study. First, among the risk factors of postoperative AKI, intraoperative MAP is particularly important, where the lowest level of MAP and duration are considered risk factors. However, no comparison can be made because of the retrospective nature of this study. Second, we analyzed the risk factors for postoperative AKI using a multivariate analysis, but this analysis was limited for adjusting the patient background characteristics. Propensity score matching would be a better analysis method for achieving statistical differences, but the study population was relatively small, and thus, this method was unsuitable. We only compared the clinical outcomes between patients with and without LVDD. However, the outcomes differ by LVDD grade. Thus, further large-scale studies with subgroup analyses by LVDD grade are needed. Finally, additional prospective studies, including those on right heart function, pulmonary vascular resistance, and pulmonary disease, which could affect CVP, are needed to confirm our results.

## Conclusion

Despite similar intraoperative and postoperative daily fluid balances, patients with LVDD have significantly increased rates of postoperative AKI and pleural effusion or edema compared to patients without LVDD. These differences might be related to fluid overload in patients with LVDD who cannot tolerate volume adjustment. Thus, individualized fluid management would be required for these patients in the perioperative period of hepatic resection.

## Data Availability

The raw data supporting the conclusions of this article will be made available by the authors, without undue reservation.
